# Too Late

**Published:** 1860-01

**Authors:** 


### TOO LATE.

Our sympathies were excited lately in receiving the following note from the only son of a mother, and she a widow who was looking up to him as the support and comfort of her old age: "Your opinion of my case only confirmed my own dread suspicions—suspicions I entertained before I wrote to you. I have very little hope left of ever being well again; but for the sake of my mother, who has grown poor, thin, and pale, as I have grown poor, thin, and pale for her sake, would I make one effort to save myself. I feel as though I should be able, were my life spared, to render her comfortable and happy for many years yet. She will be alone, indeed, when I am gone."

This noble-hearted son lost his health not from any necessity, but from want of a little knowledge as to the means of taking care of the health, such knowledge as one year's reading of this journal would have clearly and abundantly given; hence our wish, irrespective of any personal advantage, and our conviction, too, that where there is one reader, there ought to be a million.

